# Evaluation of in-vitro Antioxidant Properties of Hydroalcoholic Solution Extracts *Urtica dioica* L., *Malva neglecta* Wallr. and Their Mixture

**Published:** 2012

**Authors:** Aytaç Güder, Halil Korkmaz

**Affiliations:** *Department of Chemistry, Faculty of Arts and Sciences, Ondokuz Mayis University, Samsun, Turkey.*

**Keywords:** Antioxidant activity, Synergist effect, Mixture, *Urtica dioica *L., *Malva neglecta *Wallr

## Abstract

The study was aimed at evaluating the antioxidant activity of hydroalcoholic solution extracts of Urtica *dioica *L. (UD), *Malva neglecta *Wallr. (MN) plants and their mixture. In this study, flower (UDF), root (UDR), seed (UDS) and leaf (UDL) parts of UD and flower (MNF) and leaf (MNL) parts of MN were used. The antioxidant properties of hydroalcoholic extracts and their mixture were evaluated using different antioxidant tests such as total antioxidant activity, reducing power, superoxide anion radical scavenging, hydrogen peroxide scavenging, free radical scavenging, and metal chelating activity for comparison. In addition, total phenolic compounds in the extracts of both plants were determined as catechin equivalent. The various antioxidant activities were compared to natural and synthetic standard antioxidants such as BHA, BHT and *α*-tocopherol. According to FTC method, the both extracts exhibited strong total antioxidant activity. At the concentration of 100 μg/mL, Hydroalcoholic extracts of UDS, UDR, UDF, UDL, MNF, MNL, and UD-MN showed 81.7%, 79.8%, 78.3%, 76.4%, 77.3%, 74.1%, and 80.7%, respectively. Comparable, 100 μg/mL of standard antioxidants BHA, BHT and *α*-tocopherol exhibited 66.2%, 70.6%, and 50.1% inhibition on peroxidation of linoleic acid emulsion, respectively. In addition, UD-MN showed strong superoxide anion radical scavenging activity comparable with UDR, UDF, UDL, MNF, and MNL. Based on the findings, plants mixture was commonly found to have synergistically higher antioxidant activity.

## Introduction

Reactive oxygen species (ROS), which comprise free radicals like superoxide anion radicals (O_2_^.-^), hydroxyl radicals (HO^.^) and non-free radical species such as H_2_O_2_ and singlet oxygen (^1^O_2_), are forms of activated oxygen (1). Furthermore, excess generation of ROS decreasing the antioxidant capacity of the organism leads to inflammation, diabetes, genotoxicity, cancer and aging (2). Those primary derivates of oxygen play an important role in mediating ROS-related effects (3).

An antioxidant defense system protects the cell against the toxic effects of oxygen which evolve the mitochondrial electron transport chain in parallel (3). ROS can easily initiate the peroxidation of the membrane lipids. The peroxidation products and their secondary oxidation products are highly reactive (4, 5). In living organisms, various ROS can be formed in different ways. These ways are classified in endogenous and exogenous sources (5, 6).

Many antioxidant compounds, naturally occurring from plant sources, have been identified as free radicals or active oxygen scavengers (7). Recently, interest has increased considerably in finding naturally occurring antioxidants for use in foods or medicinal materials to replace the synthetic antioxidants, which are being restricted due to their side effects (8).

UD is a plant which belongs to the plant family of Urticaceae. Its seeds are widely used in folk medicine in many parts of Turkey, especially in the therapy of advanced cancer patients (9). Leaves of this plant have been reported to show hypotensive (10) and anti-inflammatory effects (11), to be useful in the therapy of prostatic hyperplasia (12, 13), to show diuretic (14) and immunomodulatory activity (15-17), to alleviate rheumatic pain (18), and to serve as an adjuvant therapeutic agent in rheumatoid arthritis (19).

MN is an annual and herbaceous plant. Malva species contain mucilage, malvin, tanen and glucose. The leaves and flowers of *M. neglecta *and some Malva species are used in traditional phytotheraphy. They are used in the treatment of cough, respiratory system and digestive system problems (9). Aerial parts of MN have anti-ulcerogenic activity (20). These species have also antioxidant properties (21). The local names of MN are Karagöz ebegümeci, Küçük ebegümeci, etc. This plant is important in Turkey and commonly used as a vegetable and medicinal plant (9).

The aim of this study was to investigate the antioxidant activity of extracts (UD and MN) and their mixture (for the synergy effect of MN) using different antioxidant tests. In this assay, we measured the content of total phenols and flavonoids in plants. The scope of the current paper is to investigate the antioxidant properties of some parts of the plants UD and MN that are traditionally used for medicinal purposes in Turkey.

## Experimental

*Chemicals*

Linoleic acid (cis-9,cis-12-octadecadienoic acid), p-nitro-blue tetrazolium chloride (NBT), 5-methylphenazinium methyl sulfate (PMS), nicotinamide adenine dinucleotide (NADH), absolute ethanol, hydrogen peroxide, 2,2-diphenyl-1-picryl-hydrazyl (DPPH^•^), polyoxyethylene sorbitan monolaurate (Tween-20), 3-(2-pyridyl)-5,6-bis(4-phenyl-sulfonic acid)-1,2,4-triazine (ferrozine), Folin and Ciocalteu’ s phenol reagent (Folin C), α-tocopherol, butylated hydroxyanisole (BHA), and trichloracetic acid (TCA) were purchased from Sigma (Sigma-Aldrich GmbH, Germany). Ammonium thiocyanate, ferrous chloride, potassium hexacyanoferrate (III), ferric chloride and butylated hydroxytoluene (BHT) were purchased from E. Merck. All other chemicals were of analytical grade and obtained from either Sigma-Aldrich or Merck.

*Plant material and extraction*

UD and MN plants were collected from the Samsun, Turkey, in July-August (2007), and authenticated by Prof. Dr. H. Güray Kutbay, Department of Biology, Faculty of Science and Arts, Ondokuz Mayis University. Then, plants were left on a bench to dry. The dried samples were chopped into small parts with a blender. All the samples were subjected to extraction using soxhlett extractor, with a mixture of ethanol and double-distilled water (4:1, 3x). Then the extracts were filtered over Whatman No.1 paper. The filtrates were frozen and lyophilized in a lyophilizator (Christ Alpha 1-2 LD Plus) at 10 μmHg pressure at - 50^o^C. The extracts of plants were placed in a plastic flask, and then kept at - 20^o^C until used.

*Total antioxidant activity determination*

Total antioxidant activity of extracts was determined according to the FTC method (22). For stock solutions, 10 mg of each extracts were dissolved in 10 mL of distilled water. A volume of 2.5 mL extract or standard solutions at the same concentration (100 μg/mL), was added to linoleic acid emulsion in phosphate buffer (0.02 M, pH = 7.0). Each solution was then incubated at 37^o^C in the dark. A mixture of 0.1 mL of this incubation solution, 0.1 mL of FeCl_2_ (0.02 M) and 0.1 mL of ammonium thiocyanate (30% w/v) was transferred to the test tubes, which containing 4.7 mL of ethanol (75% w/v). Then, these solutions were incubated for 3 min. Finally, the peroxide level was determined through reading the absorbance at 500 nm in a spectrophotometer (Unicam UV2-100). This step was repeated every 10 h until the control reached its maximum absorbance value. All data on total antioxidant activity are the average of duplicate analyses. The inhibition of lipid peroxidation in percentage was calculated through the following equation:

%Inhibition = [1 - (*A*_s_/*A*_c_)] × 100

Here, *A*_c_ is the absorbance of control reaction which contains only linoleic acid emulsion and phosphate buffer and *A*_s_ is the absorbance of samples (extracts) or standards (23).

*Ferric ions (Fe* ^3+^ *) reducing antioxidant power assay (FRAP)*

Reducing the power of extracts was determined according to the method of Oyaizu (24). The different concentrations (50-250 μg/mL) of extracts were mixed with 2.5 mL of phosphate buffer (0.2 M, pH = 6.6) and 2.5 mL of potassium hexacyanoferrate (III) (1%). The mixture was incubated at 50^◦^C for 20 min. A portion (2.5 mL) of TCA (10%) was added to the mixture, which was then centrifuged for 10 min at 3000 rpm (MSE Mistral 2000, UK). The upper layer of solution (2.5 mL) was mixed with 2.5 mL of distilled water and 0.5 mL of FeCl_3_ (0.1%). The Fe^3+^/Fe^2+^ transformation was investigated in the presence of extracts or standards and the absorbance values were measured at 700 nm in a spectrophotometer.

Reducing Power (%) = (*A*_s_ / *A*_c_) × 100

Here, *A*_c_ is the absorbance of control (L-ascorbic acid) and *A*_s_ is the absorbance of samples (extracts) or standards.

*Superoxide anion radical scavenging activity*

Superoxide anion scavenging activity of extracts was determined according to the method of Liu (25). In this experiments, the superoxide radicals were produced in 3 mL of Tris-HCl buffer (16 mM, pH = 8.0) containing 1 mL of NBT (50 μM) solution, 1 mL of NADH (78 μM) solution and 1 mL of extract solutions (100 μg/mL) were mixed. The reaction was started by adding 1 mL of PMS solution (100 μM) to the mixtures. The reaction mixtures were incubated at 25^o^C for 5 min and the absorbance was measured at 560 nm in a spectrophotometer. The inhibition was calculated from the following formula:

Inhibition of superoxide anion generation (%) = [1 - (*A*_s_ / *A*_c_)] × 100

Here, *A*_c_ is the absorbance of the control, and *A*_s_ is the absorbance of samples (extracts) or standards (26).

*Hydrogen peroxide scavenging activity*

The activity of the extracts to scavenge the hydrogen peroxide was determined according to the method of Ruch (27). Extract solutions (100 μg/mL) were added to 0.6 mL of hydrogen peroxide solution (40 mM, in phosphate buffer with pH of 7.4). Hydrogen peroxide concentration was determined after 10 min against a blank solution with measurement of absorbance at 230 nm in a spectrophotometer. The percentage of scavenging the hydrogen peroxide of extracts and standards were calculated using the following equation:

Scavenged % (H_2_O_2_) = [1 - (*A*_s_/*A*_c_)] × 100

Here, *A*_c_ is the absorbance of the control and *A*_s_ is the absorbance in the presence of the samples (extracts) or standards. Triplicate samples were run for each set and averaged.

*Free radical scavenging activity*

The free radical scavenging activity of extracts was measured by DPPH^•^ using the method of Shimada (28). 1 mL of DPPH^•^ solution (0.2 mM, in ethanol) was added to 3 mL of extract solutions at different concentrations (50-250 μg/mL). The mixtures were shook forcefully and allowed to stand at room temperature for 30 min. Then the absorbance was measured at 517 nm in a spectrophotometer. Control tube (containing no sample or standard) was also noted like these of samples.

Free Radical Scavenging Activity % (DPPH^•^) = [1 - (*A*_s_ / *A*_c_)] × 100

Here, *A*_c_ is the absorbance of the control and *A*_s_ is the absorbance of the samples (extracts) or standards. 

*Metal chelating activity*

Metal chelating of ferrous ions by the extracts and standards was estimated through the method of Dinis (29). A volume of 0.1 mL FeCl_2_ solution (2 mM) was added to 5 mL of extract solutions at different concentrations (50-250 μg/mL). The reaction was started via the addition of 0.2 mL ferrozine (5 mM) and the mixtures were shaken forcefully and left standing at room temperature for 10 min. The absorbance was measured at 562 nm in a spectrophotometer. The control contained FeCl_2_ and ferrozine with complex formation molecules.

Inhibition (%) = [1 - (*A*_s_/*A*_c_)] × 100

Here, *A*_c_ is the absorbance of the control and *A*_s_ is the absorbance of the samples (extracts) and standards. Triplicate samples were run for each set and averaged.

*Determination of total phenolic compounds*

Total phenolic compounds were determined with Folin C reagent according to the method of Slinkard and Singleton (30). The solutions of extract (1000 μg/mL) were prepared and each solution was diluted with 46 mL of distilled water. A volume of 1 mL Folin C reagent was added and the content of the flask mixed thoroughly. After 3 min, 3 mL of Na_2_CO_3_ (2 %) was added and then allowed to stand for 2 h with intermittent shaking. The absorbance was measured at 760 nm in a spectrophotometer. The total phenolic compounds determined as microgram of catechin equivalent that was obtained from standard graph (*r*^2^ = 0.9941).

*Determination of total flavonoid contents*

The total flavonoid was determined according to the colorimetric method (31). First of all, each powder product (0.1 g) derived from lyophilized plant extracts was dissolved in 0.1 mL of ethanol. This solution was placed in a 10 mL volumetric flask. Double-distilled water (ddH_2_O) was added to make 5 mL and 0.3 mL of 20% NaNO_2_ was added. Then, 3 mL of 10% AlCl_3_.6H_2_O was added 5 min later. After 6 min, 2 mL of NaOH (1 M) was added and the total volume was made up to 10 mL with ddH_2_O. The solution was mixed well again and the absorbance was measured at 510 nm in a spectrophotometer. The total flavonoid contents determined as microgram of catechin equivalent that was obtained from standard graph (*r*^2^ = 0.9978).

*Statistical analysis*

Experimental results were given as mean ± SD of the three parallel measurements. The experimental values were evaluated using the one-way analyses of variance (Tukey test). P-values < 0.01 were regarded as very significant and p-values < 0.05 were regarded as significant. Both operations were done with SPSS 11.5.

## Results and Discussion

*Total antioxidant activity*

Hydroalcoholic solution extracts of UD and MN plants and their mixture demonstrated antioxidant activity. Antioxidant activities were determined through ferric thiocyanate (FTC) method. This method measures the amount of peroxide produced during the initial stages of oxidation which is the primary product of lipid oxidation. In this assay, the hydroperoxide produced through the linoleic acid which was added to the reaction mixture that had been oxidized by air during the experimental period, was indirectly measured. Ferrous chloride and thiocyanate reacted with each other to produce ferrous thiocyanate by means of hydroperoxide (32).

**Figure 1:**
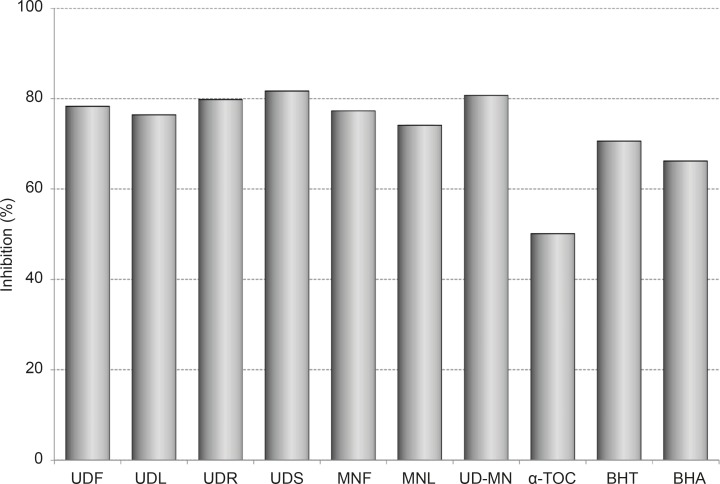
Percentage of the inhibition of lipid peroxidation through hydroalcoholic solution extracts of plants and BHA, BHT, *α*-TOC at the same concentration (100 μg/mL) at 120 h. UDF: (*Urtica dioica *L. flower), UDL: (*Urtica dioica *L. leaf), UDR: (*Urtica dioica *L. root), UDS: (*Urtica dioica *L. seed), MNF: (*M. neglecta *Wallr. flower), MNL: (*M. neglecta *Wallr. leaf), UD–MN: (*Urtica dioica *L.–*M. neglecta *Wallr.) BHA (Butylated hydroxyanisole), BHT: (Butylated hydroxytoluene), TOC: (*α*–Tocopherol).

The results of activity assays, after 100 h of incubation with linoleic acid emulsion are also summarized as inhibition % in Figure 1. 

**Figure 2:**
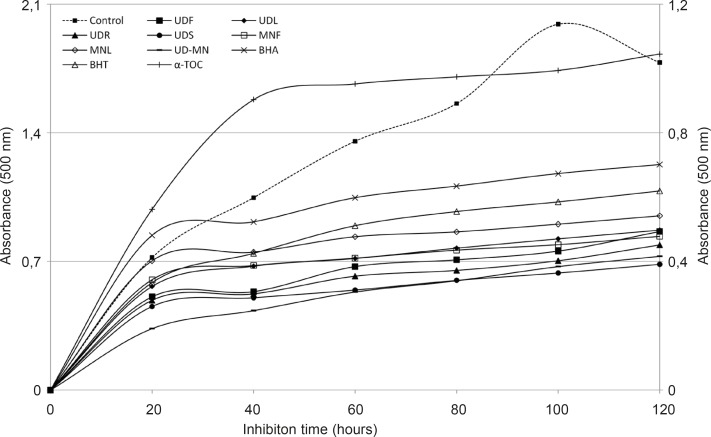
Total antioxidant activities of hydroalcoholic solution extracts of plants and BHA, BHT, *α*–TOC in the linoleic acid emulsion system were observed through the FTC method. UDF: (*Urtica dioica *L. flower), UDL: (*Urtica dioica *L. leaf), UDR: (*Urtica dioica *L. root), UDS: (*Urtica dioica *L. seed), MNF: (*M. neglecta *Wallr. flower), MNL: (*M. neglecta *Wallr. leaf), UD–MN: (*Urtica dioica *L.–*M. neglecta *Wallr.) BHA (Butylated hydroxyanisole), BHT: (Butylated hydroxytoluene), TOC: (*α*–Tocopherol). Left axis demonstrates the absorbance of control and right axis is for samples and standards

The oxidation of linoleic acid was inhibited through the tested extracts except for the extracts in comparison with the control (Figure 2).

 The different extracts showed higher antioxidant activities than BHA, BHT, and *α*-tocopherol. UDS has the most effective inhibition in this part (81.7 %). The extracts of root, flower and leaf of UD exhibited the potent antioxidant activities with 79.8%, 78.3% and 76.4% of inhibition, respectively. The extracts of flower and leaf of MN demonstrated antioxidant activities with 77.3% and 74.1% inhibition, respectively. UD-MN mixture has synergistically stronger antioxidant activity (80.7%) than both parts of MN and the root, flower, and leaf parts of UD as clearly seen in Figure 1. However, the same concentration of BHA, BHT and *α*-tocopherol inhibited lipid peroxidation up to 66.2%, 70.6% and 50.1%, respectively.

*Reducing power*

**Figure 3:**
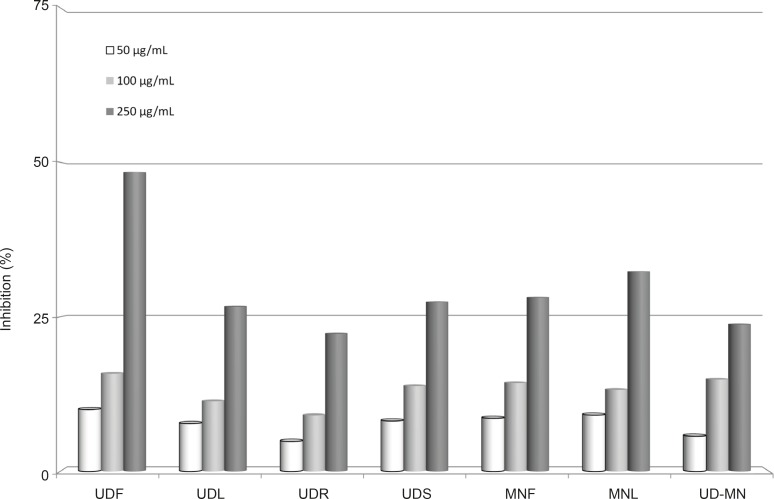
Reducing power of different concentrations (50-250 μg/mL) hydroalcoholic solution extracts of plants by Oyaizu method. UDF: (*Urtica dioica *L. flower), UDL: (*Urtica dioica *L. leaf), UDR: (*Urtica dioica *L. root), UDS: (*Urtica dioica *L. seed), MNF: (*M. neglecta *Wallr. flower), MNL: (*M. neglecta *Wallr. leaf), UD–MN: (*Urtica dioica *L.–*M. neglecta *Wallr.).

Figure 3 shows the reductive capabilities of extracts compared to BHA, BHT and α-tocopherol. The reducing capacity of a compound may serve as a significant indicator of its potential antioxidant activity (33). Reducing power of extracts exhibited the following order: UDF > MNL > MNF > UDS > UDL > UD–MN > UDR. Like the free radical scavenging and metal chelating activities, reductive potential was increased with increasing concentration. There are a number of assays designed to measure the overall antioxidant activity or reducing potential, as an indication of host’s total capacity to withstand the free radical stress (34). FRAP assay takes advantage of an electron transfer reaction in which a ferric salt is used as an oxidant (35). In this assay, the yellow color of the test solution changes to various shades of green and blue, depending on the reducing power of antioxidant samples. The reducing capacity of a compound may serve as a significant indicator of its potential antioxidant activity.

*Superoxide anion radical scavenging activity*

**Figure 4:**
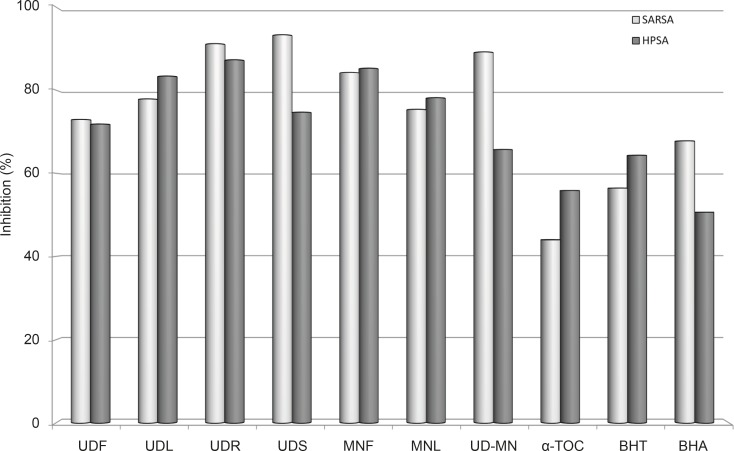
Superoxide anion radical scavenging activities (SARSA) and H_2_O_2_ scavenging activities (HPSA) at the same concentration (100 μg/mL) of hydroalcoholic solution extracts of plants and BHA, BHT, and *α*-TOC. UDF: (*Urtica dioica *L. flower), UDL: (*Urtica dioica *L. leaf), UDR: (*Urtica dioica *L. root), UDS: (*Urtica dioica *L. seed), MNF: (*M. neglecta *Wallr. flower), MNL: (*M. neglecta *Wallr. leaf), UD–MN: (*Urtica dioica *L.–*M. neglecta *Wallr.) BHA (Butylated hydroxyanisole), BHT: (Butylated hydroxytoluene), TOC: (α–Tocopherol).

In the PMS–NADH–NBT system, the superoxide anion derived from dissolved oxygen through the PMS–NADH coupling reaction reduces the NBT (3). Figure 4 presents the superoxide anion radical scavenging activity of the extracts and is compared with the same dose of known antioxidants BHA, BHT, and α-tocopherol. All of the extracts had strong superoxide anion radical scavenging activity and showed higher superoxide anion radical scavenging activity than the standard antioxidants. Except for the UDS and UDR, UD–MN mixture extract has more effective activity compared to many extracts of plant parts as well as standards (p < 0.05). Superoxide radical scavenging activity of 100 μg/mL concentration of those samples followed this order: UDS (93.3%) > UDR (91.1%) > UD–MN (89.2%) > MNF (84.2%) > UDL (77.8%) > MNL (75.4%) > UDF (72.9%) > BHA (67.8%) > BHT (56.4%) > *α*-tocopherol (44.0%). These results indicated that UD-MN have a conspicuous effect on the scavenging superoxide anion radical. UD may have biological significance in the elimination of reactive free radicals. The active components of this plant can regulate some antioxidant enzymes such as catalase, superoxide dismutase, glutathione peroxidase, and glutathione reductase in scavenging reactive free radicals. Rheumatoid arthritis, reperfusion injury, cardiovascular disease, immune injury, and cancer are related to antioxidant enzymes, which have an important role in retaining the physiological levels of superoxide, hydroxyl, alkoxyl and peroxyl radicals (36). 

*Hydrogen peroxide scavenging activity*

The scavenging ability of extracts on H_2_O_2_ is shown in Figure 4 and compared with that of BHA, BHT and α-tocopherol as standards. Extracts were capable of scavenging H_2_O_2_ in a concentration-dependent manner. Like the superoxide anion radical scavenging activity, the H_2_O_2_ scavenging activity of extracts showed higher H_2_O_2_ scavenging activity compared to the standard antioxidants at the same dose. On the other hand, UD-MN is not as effective as other parts of extracts, but UD-MN has stronger H_2_O_2_ scavenging activity when compared to standard compounds (p < 0.01). The H_2_O_2_ scavenging activity of 100 μg/mL concentration of the extracts of both plants and standards is decreased in the following order: UDR (87.3%) > MNF (85.3%) > UDL (83.3%) > MNL (78.1%) > UDS (74.7%) > UDF (71.8%) > UD-MN (65.7%) > BHT (64.4%) > *α*-tocopherol (55.9%) > BHA (50.7%). H_2_O_2_ itself is not very reactive, but it can sometimes be toxic to the cell as it may give rise to .OH in the cells (37).

*Free radical scavenging activity*

**Figure 5:**
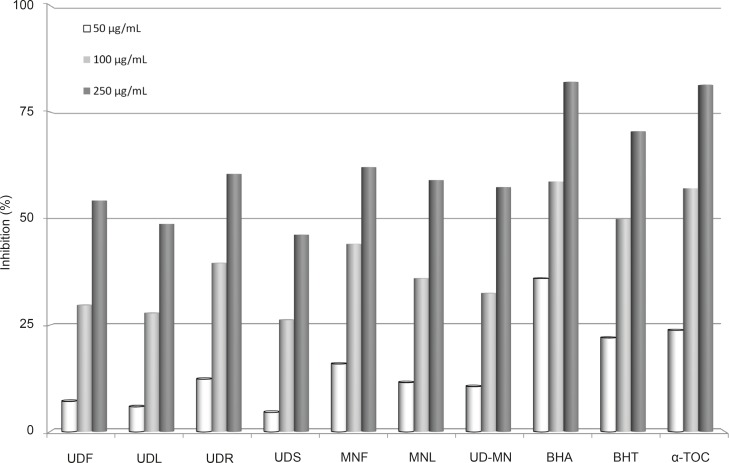
Comparison of DPPH radical scavenging activities at different concentrations (50-250 μg/mL) of hydroalcoholic solution extracts of plants and BHA, BHT, and *α*-TOC. UDF: (*Urtica dioica *L. flower), UDL: (*Urtica dioica *L. leaf), UDR: (*Urtica dioica *L. root), UDS: (*Urtica dioica *L. seed), MNF: (*M. neglecta *Wallr. flower), MNL: (*M. neglecta *Wallr. leaf), UD–MN: (*Urtica dioica *L-M*. neglecta *Wallr.) BHA (Butylated hydroxyanisole), BHT: (Butylated hydroxytoluene), TOC: (*α*-Tocopherol).

The effect of antioxidants on DPPH^•^ radical scavenging is thought to be due to their hydrogen or electron donating abilities. DPPH^•^ is a stable free radical. To become a stable diamagnetic molecule, it accepts an electron or a hydrogen radical (38). Figure 5 illustrates a significant (p < 0.05) decrease in the concentration of DPPH^•^ due to the scavenging ability of extracts and standards. All amounts of UD-MN showed higher activities than those of control and these differences were statistically very significant (p < 0.01). The scavenging effects of extracts and standards on the DPPH^•^ was decreased in the following order: BHA > α-tocopherol > BHT > MNL > UDS > MNF > UD–MN > UDL > UDF > UDR, which were 82.0, 81.4, 70.5, 62.1, 60.5, 59.0, 57.3, 54.2, 48.7, and 46.2%, respectively (250 μg/mL). In its radical form, DPPH^• ^has been disappeared on reduction by an antioxidant compound or a radical species to become a stable diamagnetic molecule resulting the color changes from purple to yellow, which could be taken as an indication of the hydrogen donating ability of the tested sample (39).

*Metal chelating activity*

**Figure 6:**
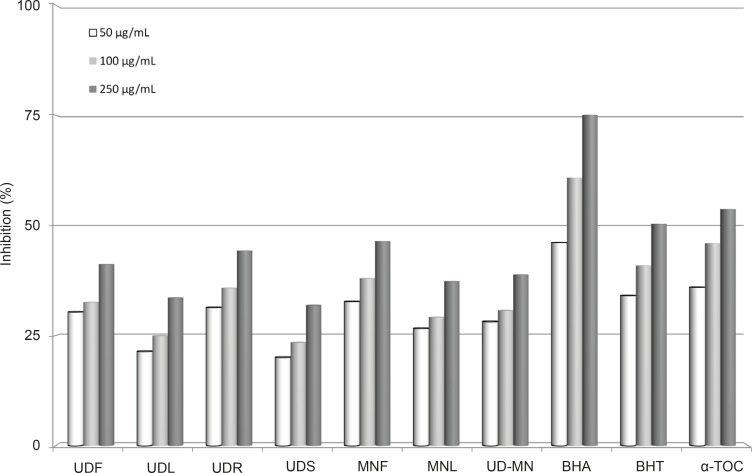
Metal chelating activities different concentrations (50-250 μg/mL) of different concentration of hydroalcoholic solution extracts of plants and BHA, BHT, and α-TOC on ferrous ions. UDF: (*Urtica dioica *L. flower), UDL: (*Urtica dioica *L. leaf), UDR: (*Urtica dioica *L. root), UDS: (*Urtica dioica *L. seed), MNF: (*M. neglecta *Wallr. flower), MNL: (*M. neglecta *Wallr. leaf), UD-MN: (*Urtica dioica *L-*M. neglecta *Wallr.) BHA (Butylated hydroxyanisole), BHT: (Butylated hydroxytoluene), TOC: (*α-*Tocopherol).

Metal chelating capacity is important since it reduces the concentration of the catalysing transition metal in lipid peroxidation (23). Ferrozine can quantitatively form complexes with Fe^+2^. In the presence of chelating agents, the complex formation is disrupted, leading to a decrease in the red color of ferrous ion and ferrozine complex. As shown in Figure 6, the formation of the ferrous ion and ferrozine complex is not complete in the presence of extracts due to the chelation of both plants with Fe^+2^. The difference between extracts of both plants and the control was statistically significant (p < 0.05). The metal chelating activity of the extracts of both plants and standards was decreased in the following order: BHA (75.1%) > *α*-tocopherol (53.7%) > BHT (50.3%) > MNF (46.4%) > UDR (44.2%) > UDF (41.2%) > UD–MN (38.8%) > MNL (37.3%) > UDL (33.6%) > UDS (31.9%). Metal chelating activity was significant since it reduced the concentration of the catalyzing transition metal in lipid peroxidation with forming *σ*-bonds with a metal, are effective as secondary antioxidants because they reduce the redox potential, thereby stabilizing the oxidized form of the metal ion (40).

*Total phenolic contents*

Phenolic compounds have antioxidant properties due to their ability of scavenging free radicals and active oxygen species such as singlet oxygen, free radicals and hydroxyl radicals (41). In our investigation, 101.1 to 213.5 μg catechin equivalent of phenols was detected in 1 mg of lyophilized plant extracts of the plants. Table 1 shows total phenols as catechin equivalent in the extracts of both plants. UD-MN has the lowest phenolic contents among the extracts given in Table 1 while they demonstrated influential antioxidant activity. Based on these results, there was no relationship between the total phenols and total antioxidant activity in extracts. The high antioxidant activity was not correlated with the phenol content owing to other factors playing major roles as antioxidants (42).

**Table 1:** Total phenol and flavonoid contents as catechin equivalent in hydroalcoholic solution extract of plants

**Extract**	**Total phenols** ^a^	**Total flavonoids** ^a^
UDF	160.6 ± 8.0	103.0 ± 5.3
UDL	132.0 ± 6.5	65.8 ± 3.5
UDR	164.0 ± 8.4	21.0 ± 0.8
UDS	213.6 ± 11.1	19.1 ± 0.9
MNF	136.1 ± 7.0	46.7 ± 2.1
MNL	106.1 ± 5.2	22.9 ± 1.2
UD-MN	101.1 ± 4.9	25.7 ± 1.1

^a^ μg/mg catechin equivalent. UDF: (*Urtica dioica *L. flower), UDL: (*Urtica dioica *L. leaf), UDR: (*Urtica dioica *L. root), UDS: (*Urtica dioica *L. seed), MNF: (*M. neglecta *Wallr. flower), MNL: (*M. neglecta *Wallr. LEAF), UD-MN: (*Urtica dioica *L.–*M. neglecta *Wallr.).

*Total flavonoid contents*

Flavonoids are very important plant constituents due to their active hydroxyl groups and antioxidant activity (43). The content of extractable flavonoid compounds in extracts, is determined from the regression equation of the calibration curve and expressed as a catechin equivalent, varied between 19.1 to 103 μg/mg lyophilized plant extracts of the plants (Table 1).

## Conclusion

Hydroalcoholic extracts of both plants showed strong antioxidant activity, reducing power, superoxide anion radical scavenging, hydrogen peroxide scavenging, free radical scavenging, and metal chelating activities when compared with natural and synthetic standard antioxidants such as BHA, BHT and *α*-tocopherol. Moreover, the obtained results of the study showed that antioxidants are efficient protective agents against the degenerative diseases and the revealed features of UD and MN may be promotive for further medicinal investigations. However, the components responsible for the antioxidant activity of extracts of both plants are currently unclear. In other words, the total antioxidant activity of MNL and MNF is almost the least whereas that of the mixture extract was higher than expected. Same case was observed in superoxide anion scavenging activity, free radical scavenging activity and metal chelating activity. As a result, it synergistically enhances the antioxidant activity to use plants mixture rather than individual plants.
